# How to measure diagnosis-associated information in virtual slides

**DOI:** 10.1186/1746-1596-6-S1-S9

**Published:** 2011-03-30

**Authors:** Klaus Kayser, Jürgen Görtler, Stephan Borkenfeld, Gian Kayser

**Affiliations:** 1 UICC-TPCC, Charite, Berlin, Germany; 2 IAT, Heidelberg, Germany; 3 IBM, Mainz, Germany; 4 Institute of Pathology, University Freiburg, Freiburg, Germany

## Abstract

The distribution of diagnosis-associated information in histological slides is often spatial dependent. A reliable selection of the slide areas containing the most significant information to deriving the associated diagnosis is a major task in virtual microscopy. Three different algorithms can be used to select the appropriate fields of view: 1) Object dependent segmentation combined with graph theory; 2) time series associated texture analysis; and 3) geometrical statistics based upon geometrical primitives. These methods can be applied by sliding technique (i.e., field of view selection with fixed frames), and by cluster analysis. The implementation of these methods requires a standardization of images in terms of vignette correction and gray value distribution as well as determination of appropriate magnification (method 1 only). A principle component analysis of the color space can significantly reduce the necessary computation time. Method 3 is based upon gray value dependent segmentation followed by graph theory application using the construction of (associated) minimum spanning tree and Voronoi’s neighbourhood condition. The three methods have been applied on large sets of histological images comprising different organs (colon, lung, pleura, stomach, thyroid) and different magnifications, The trials resulted in a reproducible and correct selection of fields of view in all three methods. The different algorithms can be combined to a basic technique of field of view selection, and a general theory of “image information” can be derived. The advantages and constraints of the applied methods will be discussed.

## Introduction

Virtual microscopy which is the work with virtual slides can be performed in two different manners: 1) interactive virtual microscopy and 2) automated virtual microscopy [1,2]. Interactive virtual microscopy translates the pathologist’s work with conventional glass slides into the digital world, and leaves all work on the microscope to the pathologist. It includes slide navigation, magnification, illumination, focus, etc. Some digital features might be added, especially the contemporary view of different slides, automated storage of areas of interest (with inbuilt expert consultation), or creation of labels. Automated virtual microscopy tries to transfer as many functions as possible to the computer with the final aim, that the system evaluates and proposes the most likely diagnoses [3-5]. Such a system must translate all items of the pathologist’s work into computerized algorithms. These have not necessarily to work in a fully compatible manner; however, they must contain modules that reflect to the corresponding pathologist’s work [6,7]. These modules will probably work in a “time sequence order”, and include in addition to statistical procedures and classifiers tools that provide a reproducible and constant image quality, object, structure, and texture related magnifications, image analysis procedures, and field of interest recognition programs.

We want to describe some basic ideas and information recognition algorithms in image analysis that can be used for field of view detection in virtual slides which is the position and size of image compartments that posses the strongest association with the underlying disease.

## Basic assumptions

The pathologist’s work is the evaluation of a diagnosis from a microscopic image, which is an image analysis algorithm in combination with external (clinical) data [1,8,9]. The pathologist’s view focuses on specific biological meaningful objects and their spatial arrangement (structure) which include a) normal objects (cells, nuclei, etc.), b) abnormal objects (cancer cells, etc.), c) external objects (bacteria, parasites, silica, etc.), d) preserved structure with abnormal cellular societies (inflammatory infiltrates, fibrosis, etc.), e) destroyed structure (granuloma, necrosis, etc.), and f) abnormal structures (adenocarcinoma, sarcomatous growth pattern) [10-13]. A diagnosis from a histological image can be evaluated by recognition and classification of the objects, the formed structures, and their spatial arrangement. It is useful to introduce different levels of structures in order to describe for example the infiltration of lymphocytes into a vascular wall (a vessel would be of higher order compared to a lymphocyte because a vessel is built by a cellular sociology including endothelial cells, smooth muscle cells, a basal membrane, etc.). The details of this concept have been described in Kayser et al. [10,14-16].

The term information is derived from the latin word informare which means “create by teaching”, in other words a communication procedure between a source (image) and the (understanding) receiver (pathologist). Shannon has analyzed the specific conditions of information transfer and content [17,18]. According to his theory information limits the broad variety of reactions of an (understanding) receiver to only one or a few appropriate ones. In other words, information is a statistical property and can be analyzed by statistical methods. Shannon introduced the term entropy as principle measure of information, which is derived from classic thermodynamics [17,18]. Entropy is a measure of the distance of a statistical population from its end stage using Kolmogorov’s axiomatic approach of non-overlapping elementary events that are characterized by a probability 0 < p < 1.

Entropies (E = ∑{pi * ln(pi)}) of different systems can be simply added (Es = ∑(Ei), if there exists no correlation between the elements of the different systems (so called strong chaos), otherwise the more general term of Tsallis entropy has to be used (Es = ∑(Eq1+Eq2) + (1-q)*Eq(1)*Eq(2)) [19-21].

## Macro- and microstages

**Figure 1:**
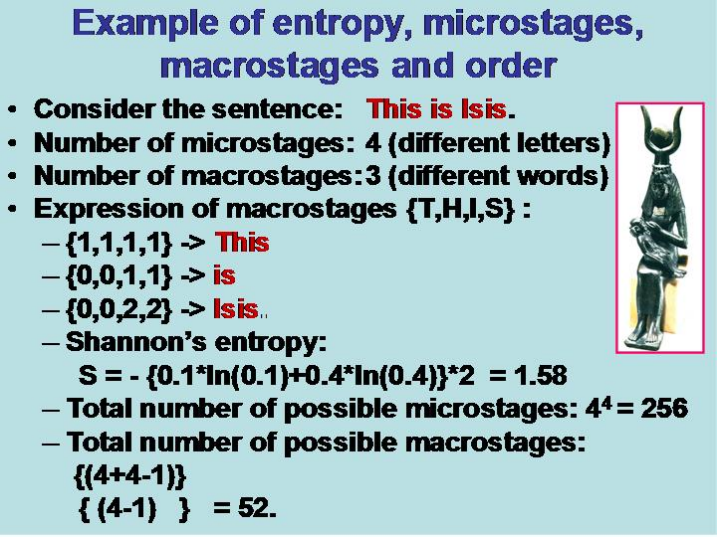
Calculation of entropy, micro and macrostages of the sentence /This is Isis/

The basic elements of a system characterized by pi might be equally distributed in the system’s space, or agglutinate to certain formations which can be considered as a “subspace”. They are called macrostages. One can define the macrostages as new (higher order) events, and calculate the entropy of the original system based upon the macrostages and their internal entropy [22,23]. The number of microstages gives the maximum number of potential macrostages. An illustrating example is shown in figure 1 which is described in detail in [22]. The letter {T,H,I,S} are the microstages, and the words {THIS, IS, ISIS} form the macrostages. The sequence of the macrostages form the structure.

The calculated Shannon entropies of the macrostages within the system (This is Isis) result in:

This : [-0.92]; is: [-0.46]; Isis: [- 0.64]; ∑ = - 2.02

that of the total system without macrostages {this is Isis} = -1.58,

and based upon the marcostages alone {[this] [is] [Isis]} = -1.08

The calculated probability of the macrostages based upon their internal entropies results in:

P(this) = (1.92)/5.02 = 0.38

P(is) = (1.46)/5.02 = 0.29

P(isis)= (1.64)/5.02 = 0.33

This is Isis: E = {0.38*ln 0.38 + 0.29*ln 0.29 + 0.33 * ln 0.33 = - 1.09

The differences between the macrostages are: [-0.46] + [+0.22] = - 0.18.

If we transform the sequence into the question:

Is this Isis? we will get: [+0.46] + [-0.28] = + 0.18.

The calculation of the total entropy of the (macrostage) system depends upon its structure, or, in other words, the calculation of macrostage entropies can be applied in relation to internal structures, such as sequential arrangement or spatial relationships [16,22,23].

### Entropy calculation in relation to histological images (virtual slides)

**Figure 2:**
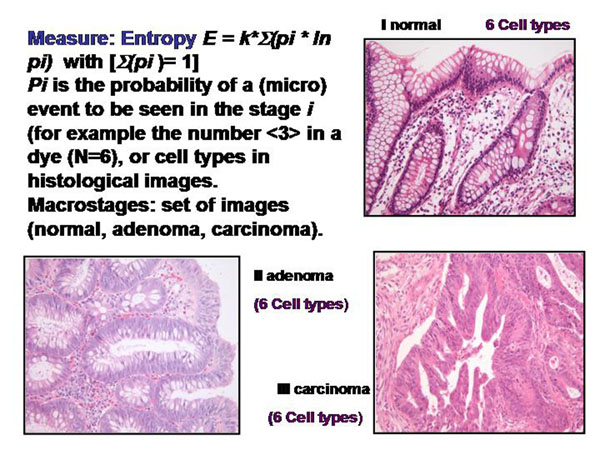
Example of cell types in histological images of colon diseases (normal, adenoma, and carcinoma) used for entropy calculation (Shannon and Tsallis)

The information of a histological image which a pathologist can derive depends on the presence and spatial arrangement of cells or nuclei respectively. The different cell types that are present in such a slide can be addressed to microstages, and the corresponding disease to macrostages respectively. The microscopic images, the associated diagnoses, and the analyzed microstages are shown in the figure 2 – figures 4. All in all 15 different cell types, and 8 different diseases are taken into account (figure 5). The assumed cellular distributions are given in figure 6, and the computed entropies are shown in figure 7. As expected, notable differences exist between the different images (diseases). They are, however, not striking between quite different diseases, for example between a small cell lung cancer and normal lung parenchyma. The computed entropies can obviously not directly be translated to the biological significance of the corresponding disease.

**Figure 3:**
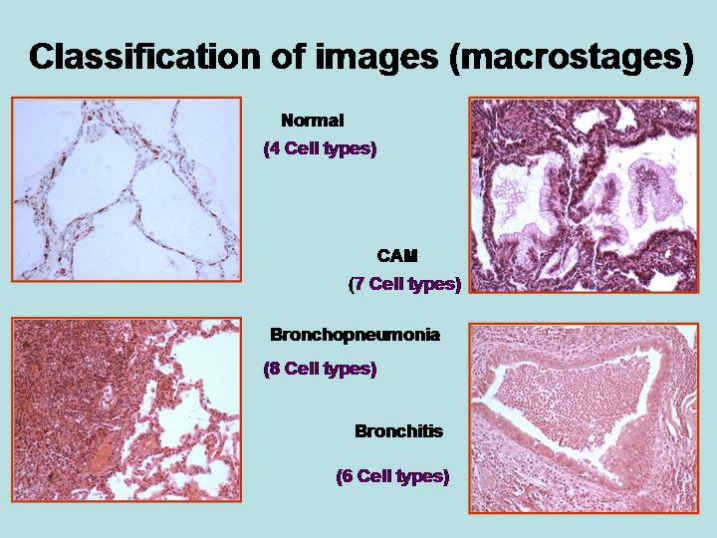
Example of cell types in histological images of lung diseases (normal, congenital adenomatoid malformation (CAM), bronchopneumonia, and bronchitis) used for entropy calculation (Shannon and Tsallis)

**Figure 4:**
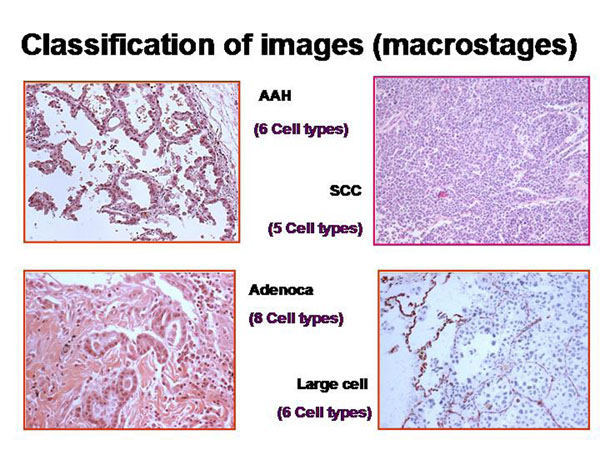
Example of cell types in histological images of lung diseases (atypical adenomatoid hyperplasia (AAH), small cell anaplystic carcinoma (SCC), Adenocarcinoma (Adenoca), and large cell anaplastic carcinoma (Large cell) used for entropy calculation (Shannon and Tsallis)

**Figure 5:**
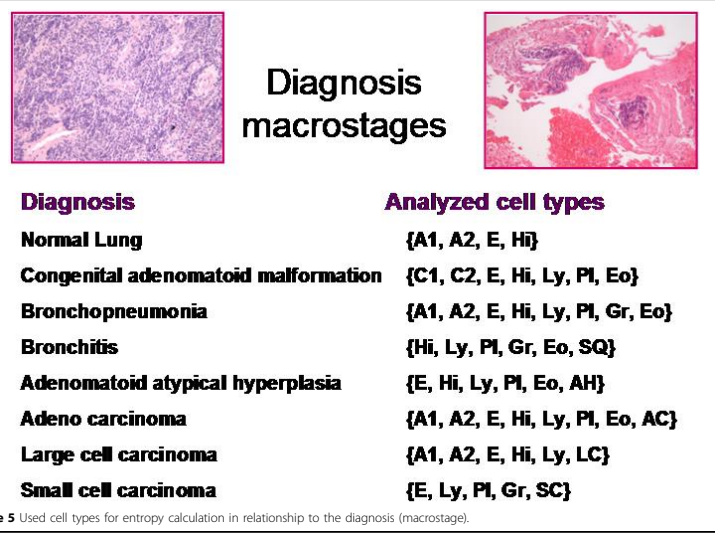
Used cell types for entropy calculation in relationship to the diagnosis (macrostage)

**Figure 6:**
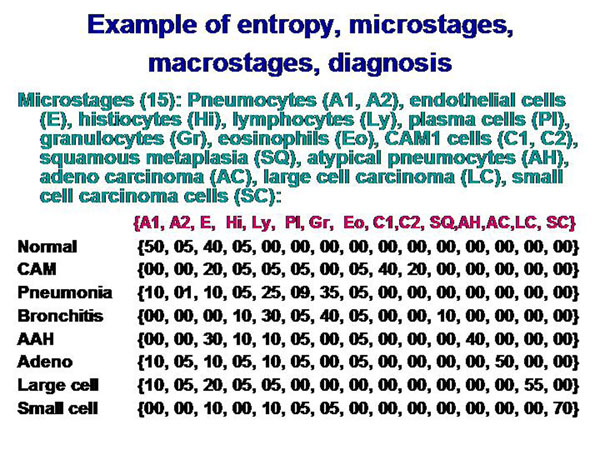
Explanation of used cell types and their assumed relative frequency in the macrostage

**Figure 7:**
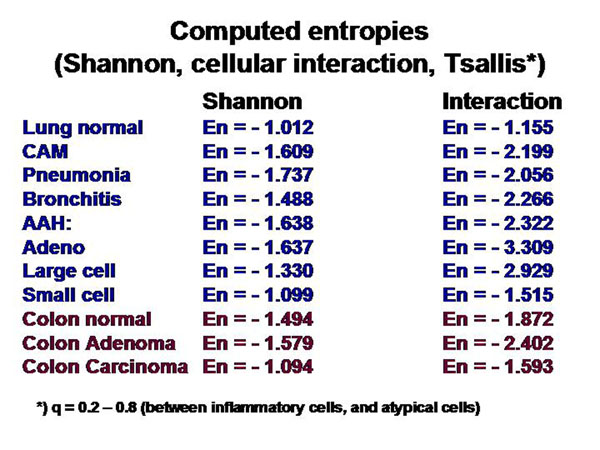
Computed entropies of the model. No direct association of amount of entropy and clinical behavior of the disease was obtained

## How to refine the entropy approach?

### Definition of image associated macro- and microstages

All (interactive) diagnostic information of a digitized image is derived from biological meaningful objects such as cells, nuclei, mitoses, vessels, etc. In other words, an analysis of the image information results in a meaning, which is a probability function of the (predefined) diagnoses and the image information. The higher the probability the more accurate is the diagnosis. The advantage of such an algorithm is the “relatively” constancy of objects (and derived information) compared to the broad variations of images belonging to the same diagnosis [3,8].

We can consider that image information is an entity that is primarily separated from the set of diagnoses. This theory induces that image information can be described as a mapping of diagnoses M(D) on the image pixels {p(x,y,g)} with

M({Di},P) -> p{px(x,y,g)} with p{px(x,y,g)} = maximum

for the (evaluated) diagnosis D.

Using the entropy approach we create a n-dimensional space of elementary image events and analyse the distribution in the different diseases or macrostages. It would be of formal advantage, if we could define certain elementary events that are independent from the associated meaning, i.e. independent from external knowledge. In fact, this is possible by application of stochastic geometry which has been described by Stoyan et al [24].

Naturally, one could use the pixels as elementary events and associated spectral functions in order to create the set of elementary events. However, this approach would leave us again with the problem of handling broad image variance and low probability levels.

The basic elements (or image primitives) can also be calculated by introduction of a (spatial) relationship function. It is usually called neighbourhood condition, such as Voronoi’s or O’Callaghan’s condition [25-27]. The simplest case is a neighbourhood function f(x,y) with

F(0,1) = 1 iff g(x,y)>threshold, and g(x+1,y)>threshold, or g(x,y+1)>threshold, i.e., two pixels are neighbors iff both of them posses a gray value above a certain predefined threshold (or within a predefined bandwidth of gray values). Naturally, a negative definition can also be applied (<threshold).

This definition allows us to define a set of primitive elements, that form an object, i.e. an elementary element of image information (object, structure, texture).

The different primitive elements include

Isolated points (i.e. pixels without neighbors)

Fibers (pixels possessing a “line” of neighboring pixels, and different start and end pixel

Circles (pixels possessing a line of neighboring pixels, and identical start and end pixel

Plateaus (a set of pixels with a number of neighboring pixels>2 and connected points or lines).

Any biological meaningful object can be broken down to a set of these four primitives; for example a membrane consists of a line or a circle, a nucleus of a circle and at least one plateau, a non-completely segmented nucleus of lines, points, and plateaus, etc. .

**Figure 8:**
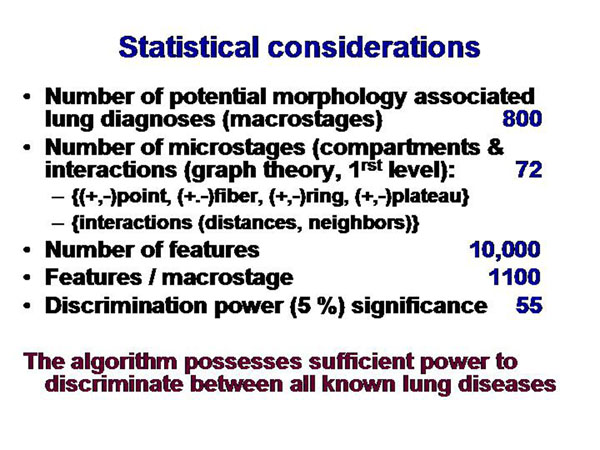
Statistical considerations on macrostages. About 800 different lung diseases with characteristic morphology are known. Only 55 of 1,100 potential features would be needed to obtain a discrimination power p > 0.95.

In potential clinical application, this approach has to work with approximately 800 different macrostages (lung diseases, derived from [28], and 10,000 different features (see figure 8). To discriminate between different macrostages with a significance of s>0.95 only 55 features per macrostage out of 1,100 available features per macrostage would be necessary.

## Implementation

**Figure 9:**
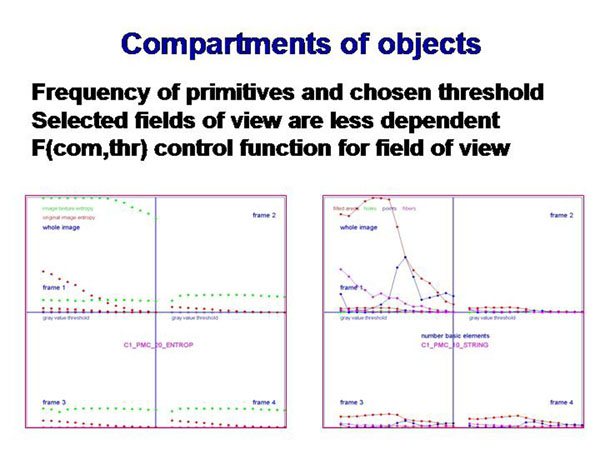
The generation of image primitives depends upon the chosen threshold. It remains nearly constant in correctly hosen fields of view, and can be a measure for information content in relation to the whole image.

The selection of an appropriate threshold and/or bandwidth of the gray values as well as the image size in pixels are parameters that influence directly the implementation of this algorithm. Therefore, it has been tested on automated selected areas of interest which have been determined by analysis of texture and object features, as described elsewhere. Within the selected areas of interest the chosen threshold is of only limited influence on the expression of the elementary primitives in contrast to the whole image (see figure 9). Thus, working in correctly selected areas of interest a threshold can be chosen within a broad range without falsifying the results. On the other hand, the described technique might be useful to check the correctness of the selected field of view. An approach to finally classify diseases by the described algorithm is in preparation.

## Discussion

The development of reliable and practice oriented scanners which scan whole glass slides has opened a new door in diagnostic surgical pathology or tissue – based diagnosis [1,3,5,6,29-31]. The mechanical and optical problems are in so far solved as the new canner generation can be successfully implemented into the workflow of routine diagnosis [9]. The next step waits for opening new and attractive functions of these systems. These will include the mandatory replacement and improvement of classic microscope handling, the implementation of new viewing and measurement functions, as well as the search for automated diagnosis systems. These will probably start with the implementation of so-called assistants that will guide the pathologist through all the possible tools. As in all such trends, the final aim would probably be an automated diagnosis system, which the pathologist has to control, and which might at a very later stage control itself.

In this article we describe only one of the possible manners to build and to implement such a system. Other algorithms have been successfully tested too [32-34]. The main idea is that we try to separate different functions that are used in the pathologist’s thinking and diagnostics, and not to be confused with the contemporary application of algorithms that are in principle separated. When in the Middle Ages some genius persons tried to directly copy the flight of birds, they failed because they did not separate the upstream forces from the velocity (forward) movement. The separation of both forces induced the successful development of airplanes that have thought to be never become reality in the past.

We have shown the reader an approach that in a similar manner separates the information given in an image, and its evaluation and interpretation based upon known classification of information (diseases) by a pathologist. Having finally tested the approach, a more generalized theory of performing information into knowledge and competence in virtual microscopy is indicated.

## Competing interests

The authors declare that they have no competing interests.

## Acknowledgement

The financial support of the Verein zur Förderung des biologisch technologischen Fortschritts in der Medizin e.V. gratefully acknowledged.

This article has been published as part of *Diagnostic Pathology* Volume 6 Supplement 1, 2011: Proceedings of the 10th European Congress on Telepathology and 4th International Congress on Virtual Microscopy. The full contents of the supplement are available online at http://www.diagnosticpathology.org/supplements/6/S1
